# Collision of Basal Cell Carcinoma with Apocrine–Sebaceous–Follicular Unit Neoplasms

**DOI:** 10.3390/dermatopathology11040032

**Published:** 2024-10-25

**Authors:** Enric Piqué-Duran

**Affiliations:** Dermatology Department, Doctor Jose Molina Orosa Hospital of Lanzarote, 35500 Arrecife, Spain; enric@aedv.es

**Keywords:** basal cell carcinoma, collision, apocrine–sebaceous–follicular unit tumors

## Abstract

Background: Tumor collision is a rare event, with an estimated incidence of 0.0017%. Seborrheic keratosis, melanocytic nevi, and basal cell carcinoma (BCC) are by far the most common entities involved in collisions. Most authors consider collision to be an incidental event. I planned a retrospective study comparing BCC/apocrine–sebaceous–follicular unit (ASFu) neoplasm collisions with squamous cell carcinoma (SCC)/ASFu neoplasm collisions. Materials and methods: Files from 2005 to 2017 from Dr. José Molina Orosa Hospital were assessed; in the review, cases of collisions between BCCs or SSCs and ASFu tumors, including cysts, were identified. Results: Out of 3247 BCC cases, 12 biopsies were retrieved. Of 825 biopsies, none belonged to the SCC group. The ASFu tumors that collided with a BCC were as follows: four hidrocystomas, three infundibular cysts, two steatocystomas, two trichilemmomas, one spiradenoma, and one clear-cell hidradenoma (one patient had two cysts associated with a BCC). These cases correspond to seven female patients and five male patients aged between 26 and 91 years old. A quarter of these patients were immunosuppressed. Most ASFu neoplasms were found to be located beneath the BCC (8/12). Discussion: To the best of my knowledge, this report describes three new collisions of BCCs with ASFu neoplasms (infundibular cysts, steatocystomas, and a spiradenoma). My results also suggest that immunosuppression could be a factor that predisposes a patient to these collisions. I review current hypotheses in an effort to explain these collisions and contribute some new theories.

## 1. Introduction

Excluding in situ squamous cell carcinoma (SCC) and its variants (actinic keratosis, Bowen’s disease, and erythroplasia of Queyrat), basal cell carcinoma (BCC) is the most frequent skin malignancy, representing 70% of cases [1]. In addition, BCC is one of the types of tumors more frequently involved in collisions. According to many studies in the literature, collisions between tumors, including BCC collisions, are incidental. 

I decided to study the collisions between BCCs and tumors of the apocrine–sebaceous–follicular unit (ASFu), including cysts, and compare them with the collisions that occur between SCCs and AFSu neoplasms. Boyd and Rappini [2] considered collisions an incidental event. They found an incidence of 0.0017% [2]. However, in my experience, the association of BCC–ASFu tumors is too frequent to be considered a random event. Because SCC is another common epithelial skin malignancy, I chose SCC–ASFu tumor collisions as a comparative group.

## 2. Material and Methods 

According to Boyd and Rappini [2] a histopathological collision is “the presence of two or more neoplasms in the same sample”. This definition includes samples that contain two neoplasms that are clearly separated. In my opinion, these cases cannot be considered collisions. For me, a collision is “the presence of two or more neoplasms in the same sample that are in contact or have a relationship”.

I reviewed dermatopathological reports for BCC and SCC cases from January 2005 to July 2017 at Doctor Jose Molina Orosa Hospital, Lanzarote (Spain), looking for collisions with ASFu tumors. The search criteria were as follows: (a) an occurrence of the collision of tumors; (b) two or more diagnoses in the same biopsy; (c) BCC or SCC with some kind of differentiation, including basosquamous tumors; (d) clear-cell BCC or clear-cell SCC; and (e) keratotic BCC. 

I excluded the following cases: (a) cases of in situ SCC, including its variants (actinic keratosis, Bowen’s disease, and erythroplasia of Queyrat); (b) cases that occurred in the context of a sebaceous nevus of Jadassohn; and (c) BCC–SCC collisions.

I reviewed hematoxylin–eosin (H&E)-stained slides in all cases. In selected cases, sections that had been stained with the avidin–biotin immunoperoxidase method using antisera against BerEP4, EMA, CEA, alpha-smooth muscle actine (SMA), cytokeratins CKA1/A3, and Ki67 (Table 1) were studied. Diaminobenzidine was used as the chromogen.

Histologically, I assessed the type of BCC according to the WHO classification (2006) [3]; additionally, I assessed the location of the ASFu tumor with respect to the main tumor (BCC or SCC) as follows: (a) above, (b) beneath, or (c) intermingled. 

From medical reports, I retrieved patient data on age, sex, immunosuppressive status, and the clinical diagnosis and evolution of the lesion. 

## 3. Results

From January 2005 to July 2017, 3247 cases of BCC and 825 cases of SCC were identified from the 23,265 skin biopsies studied at Doctor José Molina Orosa Hospital, Lanzarote (13.96% and 3.55%, respectively). The ratio of BCC to SCC was approximately 4:1. 

For BCC, I retrieved 23 biopsies that I suspected to be cases of collision with ASFu tumors. After the review, I excluded 11 for the following reasons: (a) if the tumors were separated and, therefore, not in collision (3 cases); (b) if, in addition to the BCC, the only finding was a foreign-body granuloma due to keratin (3 cases); and (c) if there were ductal or follicular dilatations, but these were not big enough for us to consider them to be cysts (5 cases). Thus, 12 biopsies were included for study.

For SCC, the single biopsy that was retrieved was excluded because there were two separate neoplasms. 

Because of the small number of cases and the fact that there were no lesions in the comparative group, no statistical methods were applied.

**Figure 1:**
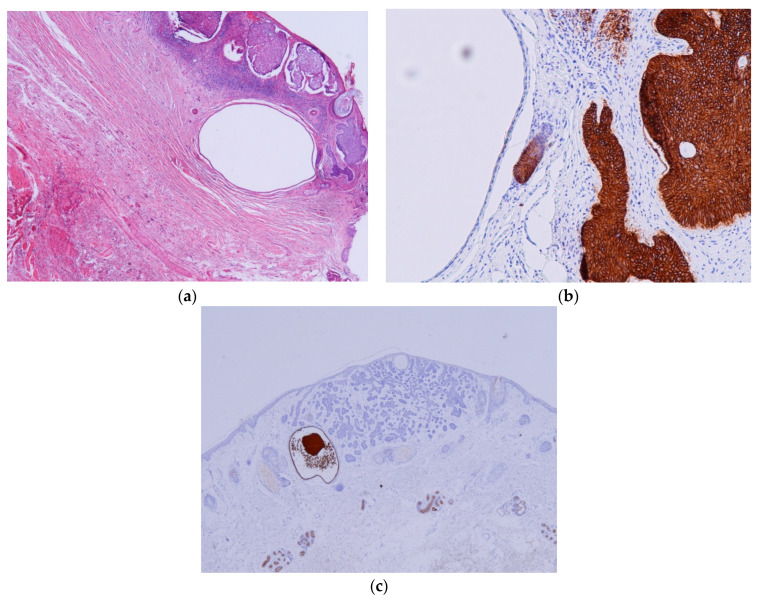
Hidrocystoma cases. (**a**) Case 1: An empty space beneath a nodular BCC corresponding to a hidrocystoma; this could be confused with a detached BCC nest., (H&E ×20). (**b**) Case 1: Conversely to BCCs, the hidrocystoma is negative for BerEP4 (BerEP4 ×100). (**c**) Case 8: CEA stain highlights hidrocystoma, while BCC remains negative (CEA ×10).

**Figure 2:**
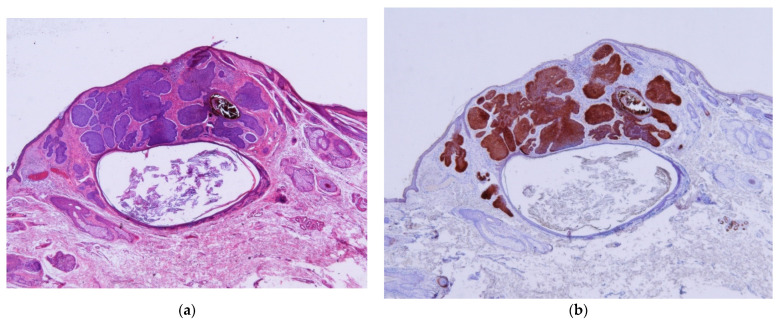
Infundibular cysts. (**a**) Case 2: A cystic structure filled with basket-woven keratin that could be confused with a keratotic BCC, corresponding to an infundibular cyst beneath a nodular BCC (H&E ×20). (**b**) BerpEP4 clearly distinguishes the infundibular cyst from the BCC (BerAp4 ×20).

**Figure 3:**
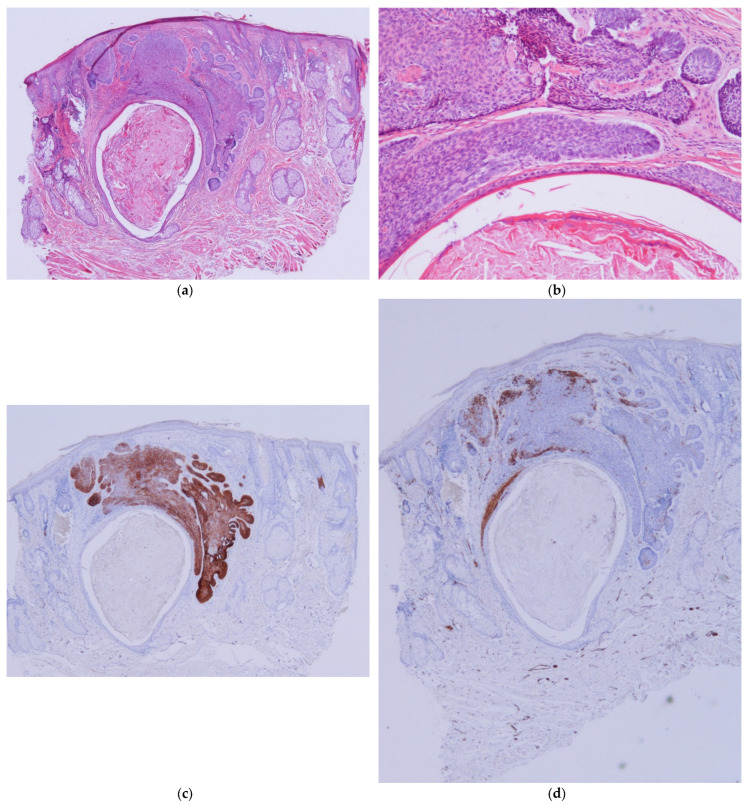
Steatocystoma cases. (**a**) Case 6: A cystic structure in connection with a sebaceous gland beneath a BCC (H&E ×20). (**b**) Case 6: Detail of a BCC in close relationship with a cystic structure, a steatocystoma, that shows a cuticula in the luminal layer. This case could be confused with necrosis in the mass inside a BCC nest (H&E ×100). (**c**) Case 6: BerEP4 stain shows positivity for BCC and negativity for steatocystoma. A sebaceous lobule is connected with the cyst. (BerEP4 ×20). (**d**) SMA shows the presence of a pili erector muscle in contact with steatocystoma. In this case, SMA shows focal positivity in BCC (Actin ×20).

**Figure 4:**
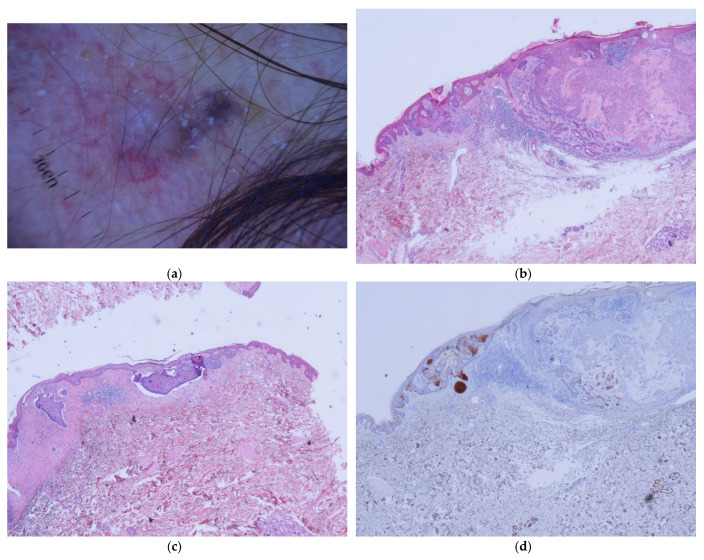
Trichilemmoma cases. (**a**) Case 10: Dermatoscopic picture; here, there are two different structures—the left one corresponds to a trichilemmoma, while the right one is a BCC. (**b**) Case 11: Around the pale squamous tumor, there is a thick basal membrane; here, the lesion corresponds to a trichilemmoma—on the left side, some small basaloid nests can be seen hanging from the epidermis, and these are superficial BCCs. Between both lesions, a reactive ductal hyperplasia exists (H&E ×20). (**c**) Case 11: Another field shows a clearer BCC in this panoramic view without a trichilemmoma (H&E ×10). (**d**) Case 11: BerEp4 highlights BCC nests (BerEP4 ×20).

**Figure 5:**
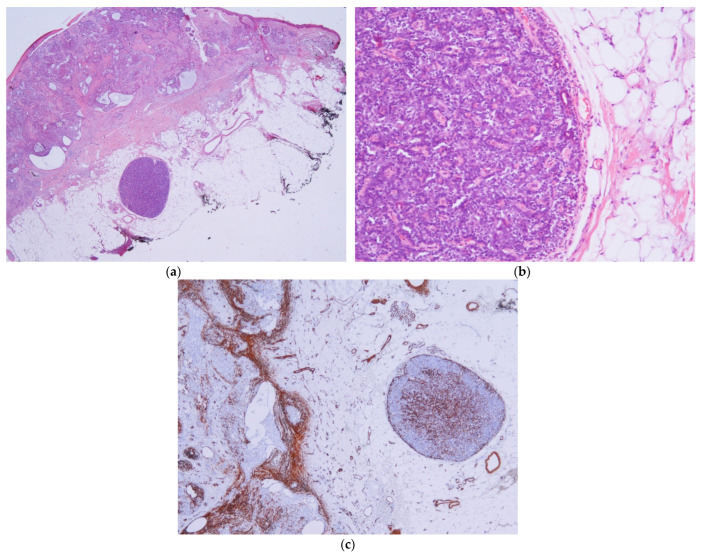
Spiradenoma. (**a**) Case 4: A basaloid nodule in the fat beneath a BCC with pseudo-cystic mucin-filled spaces (H&Ex10). (**b**) Case 4: Detail of the spiradenoma; here, a basaloid nodule with basal membrane and lymphocytes is intermingled with the tumoral cells (H&E ×40). (**c**) Case 4: Spiradenoma shows SMA positivity in the center and the peripheral layer, while SMA stains the stroma and focal areas of the BCC (SMA ×20).

**Figure 6:**
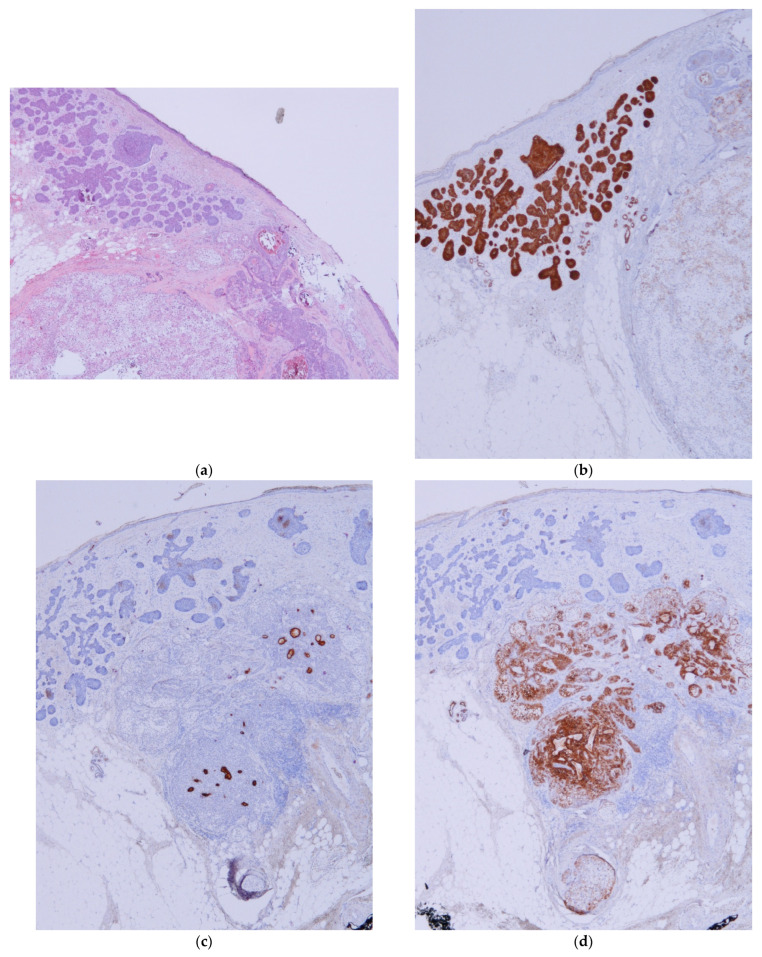
Clear-cell hidradenoma. (**a**) Case 9: A clear-cell hidradenoma on the right, composed of different types of cells; in the picture, clear cells predominate, while nests of squamoid cells that contain some cystic structures are closer to the epidermis; on the left side, there is a micronodular BCC (H&E ×20). (**b**) Case 9: BerEP4 highlights BCC. The clear cells of the clear-cell hidradenoma are weakly positive (BerEp4 ×20). (**c**) Case 9: The presence of ducts is demonstrated by CEA (CEA ×20). (**d**) Case 9: The squamoid cells show positivity with EMA stain (EMA ×20).

BCC–ASFu collisions comprised 0.05% of all biopsies and 0.37% of all BCCs. The neoplasms that accompanied the BCCs were as follows: four hidrocystomas (Figure 1), three infundibular cysts (Figure 2), two steatocystomas (Figure 3), two trichilemmomas (Figure 4), one spiradenoma (Figure 5), and one clear-cell hidradenoma (Figure 6). In Case 7, the BCC collided with both an infundibular cyst and an apocrine hidrocystoma. Most of the cases occurred in patients’ heads (9/12), while the rest occurred in their trunks (3/12). In 8 out of 12 cases, the ASFu neoplasm was located beneath the BCC. In the other four cases—which included the trichilemmoma cases, the clear-cell hidradenoma cases, and Case 7—both neoplasms were intermingled. Nodular BCC was the most common type of BCC. Curiously, the two cases of trichilemmomas had collided with superficial BCCs. 

The included series of cases comprised seven female patients and five male patients, with a mean age of 67 years (age range of 26–91 years). In all cases, the clinical diagnosis was BCC. Interestingly, three cases presented in an immunosuppression state. In addition, the patient of Case 2 developed lung cancer shortly after the diagnosis of the collision, so four cases were considered to be immunosuppressed. Table 2 and Table 3 show the clinical and histopathological features of my series, respectively.

In all of the cases, the immunohistochemistry process was useful in distinguishing the BCC from the ASFu neoplasm. Table 4 shows the results of the immunohistological studies. BerEP4 was positive in all BCC cases and negative for the ASFu neoplasms, except for the clear cells of the clear-cell hidradenoma and focally in the trichilemmomas, which showed positivity. Interestingly, EMA was particularly useful in distinguishing clear-cell hidradenoma (+) from of BCC (−). In addition, CEA clearly differentiates hidrocystomas—which show positivity for the luminal layer—from BCCs. Curiously, SMA was positive in five out of seven cases of BCC; of these cases, Case 7 showed diffuse positivity, whereas the remaining cases presented with a focal pattern. In addition, the stroma was focally positive in three cases (Table 4).

## 4. Discussion

Tumor collision is a rare event, with an incidence of 0.0017% [2]. However, this incidence level is probably underestimated. In this study, the incidence of collisions was 0.05%, and this was calculated only for BCC and ASFu neoplasms. This discordance could be due to dermatopathologists diagnosing a main tumor and paying no attention to secondary neoplasms. Seborrheic keratosis, melanocytic nevus, and basal cell carcinomas are by far the most frequently involved tumors. This is probably because they are the most commonly biopsied tumors.

BCCs have been observed to collide with seborrheic keratosis [4], melanoma [5,6], melanocytic nevus [4], squamous cell carcinoma [7,8,9], neurofibroma [4], and leiomyosarcoma [10]. For ASFu neoplasms, BCC has been found to have collided with sebaceous carcinoma [11], trichofolliculoma [12], trichoepithelioma [13,14,15], hidrocystoma [16], and trichilemmoma [17,18]. The BCC-cylindroma collision described by Sham et al., in my opinion, corresponds to a BCC with a conspicuous interspersed basal membrana [19]. Thus, some of the tumors described in this series (infundibular cyst, steatocystoma, and spiradenoma) have not been reported previously. Case 9, with BBC–clear-cell hidradenoma collision, has been previously reported by us [20]. 

On the other hand, SCC has been observed in collision with in situ melanoma [21] and BCC [7,8]. To the best of my knowledge, no collisions have been described between SCC and ASFu neoplasms.

Some reports describe a BCC or an SCC that appear from the wall of an infundibular cyst [22,23] or a hidrocystoma [24]; however, these cases are not real collisions. They have to be considered to be cases of malignant transformations of cysts. Conversely, in my series, these cases show two independent lesions without transitions occurring between them; therefore, these are true collisions.

Currently, most authors consider collisions to be incidental events; however, some theories have been proposed to explain collisions [25]. These include: (a) a coincidental incidence of two common entities; (b) a common trigger such as actinic damage that is related to BCC, SCC, and melanoma, which may favor the occurrence of collisions among them; (c) the induction of one neoplasm from the stroma may lead to a predisposition to the occurrence of a new neoplasm, in a similar way to the process that occurs with dermatofibromas [4,26]; and (d) the presence of a hybrid neoplastic cell that has retained properties from different cell lines. However, I propose some new factors that may influence the occurrence of collisions: (1) Most authors believe that BCCs derive/differentiate from follicular germinative cells; hence, a common embryological origin of ASFus could explain the results of this study [27,28]. (2) According to my results, a state of immunosuppression could favor the occurrence of collision; of my cases, 25–33% were immunosuppressed. (3) Finally, a common genetic or molecular basis may promote the development of some different tumors. BCCs may share genetic alterations with some ASFus in this case [29,30].

The diagnosis of BCC–ASFu neoplasm collision is a real challenge. On one hand, BCCs can show differentiation toward some structures of ASFus; on the other hand, ASFu neoplasms usually have a basaloid appearance. 

Keratotic and infundibulocystic variants of BCCs contain cystic structures. In these cases, the walls of the cyst are BCCs, and the cysts are usually numerous and small. This is unlike cases of BCC–infundibular cyst collision, in which there is only one large cyst with a wall that is not a BCC. BerEP4 easily distinguishes between these entities (Figure 2). 

Hidrocystoma and steatocystoma are presented as an empty space surrounded by a thin wall. In the context of a BCC, this can easily be confused with an area of necrosis in the mass, especially in superficial biopsies [31]. In steatocystomas, the presence of an eosinophilic hyalinized cuticle and sebaceous lobules in the cyst wall help to differentiate a collision from a BCC with necrosis in the mass. Hidrocystomas may exhibit decapitation secretion; however, if this is absent, it may be difficult to notice the presence of an hidrocystoma in the context of a BCC. Again, immunochemistry (especially BerEP4, CEA, and SMA) can be used to easily identify an hidrocystoma (Figure 1) or a steatocystoma (Figure 3) in the context of a BCC.

Curiously, most of the BCC collisions in these samples occurred with cysts, which were located beneath the BCC. Although it is tempting to consider strangulation to be the etiopathogenic mechanism of this collision, it is hard to sustain this theory when I consider the fact that only 7 out 3247 BCC biopsies showed this association. 

Interestingly, the two trichilemmoma–BCC collisions (Figure 4) included in this study, in addition to the three cases described in the literature, occurred with the superficial variant of BCC [17,18]. Crowson et al. [18] have described four cases of this collision; two of them associated trichilemmomas with superficial BCC, while the rest were described by the authors as nodular BCC and atypical basaloid proliferation. In my opinion, both lesions correspond to trichilemmomas with basaloid differentiation [32]. We have no explanation for this association.

Currently, spiradenoma is considered by most authors to be apocrine in origin [33]. In this case, the spiradenoma was located in the subcutis beneath the BCC. Because spiradenoma is a basaloid nodule, it can be easily confused with a deep BCC nodule. Nevertheless, the presence of a basal membrane and lymphocytes intermingled with basaloid cells, in addition to the cytologic differences and stroma, should alert us to the fact that there are two different neoplasms. The BerEp4 negativity and CEA positivity in the spiradenoma confirm the fact that a real collision occurred (Figure 5). An expected finding was SMA positivity in the outer layer and a delicate reticulate pattern in the center of the spiradenoma. Curiously, SMA marked the stroma and some nests of the BCC. Despite the SMA positivity in both tumors, they had different patterns that helped to distinguish both lesions in this case.

Clear-cell hidradenoma, also known as apocrine hidradenoma, solid cystic hidradenoma, or nodular hidradenoma, usually comprises four types of cells (clear or pale cells, plasmacytoid or polygonal cells, mucinous cells, and squamoid cells) in a variable proportion and in a nodular or multinodular solid or solid–cystic pattern. In this case, clear cells can be misinterpreted as focal areas of clear-cell BCC, the squamoid cells can be misinterpreted as areas of keratotic differentiation of the BCC, and cystic areas can be misinterpreted as necrosis in the mass; however, the sharp demarcation of the clear-cell hidradenoma, and the different patterns of the tumors (nodular in the clear-cell hidradenoma and micronodular in the BCC), allow us to diagnose the occurrence of a collision. In addition, the stromas are quite different, indicating the presence of sclerosis in the clear-cell hidradenoma and the presence of mast cells and numerous fibrocytes in the BCC. Again, the immunochemistry process was used to clearly differentiate both tumors (Figure 6).

Curiously, SMA showed positivity in five out of seven BCC cases, with either tumoral cells or stroma cells, or both. The SMA positivity has been interpreted as myoepithelial differentiation of the BCC [34]. Nevertheless, according to the series from Tsukamoto et al., SMA was found to be positive in over 50% of BCCs [35]. Thus, SMA positivity in BCC is perhaps a common finding [35].

This study has some limitations. Despite the fact that my files contained 23,265 biopsies, only 12 BCC collisions were retrieved, and there were no SCC collisions. I was therefore unable to compare both groups and so the conclusions have to be considered with caution.

Although this is a retrospective study, most cases were diagnosed recently. This could be because my interest in collisions has increased since 2010, when we presented a series in a meeting [7]. This may imply that in order to diagnose collisions, dermatopathologists must be alert to the occurrence of this event.

Other difficulties were encountered. For example, I faced difficulties when attempting to classify BCCs according to the WHO’s classification, because, in real life, many BCCs appear to be mixed and sometimes show many different patterns. I considered the predominant patterns. In addition, many BCCs contain ductal dilatation. The differentiation between cysts and ductal differentiation is subjective. To the best of my knowledge, there are no size criteria for distinguishing both. I consider the presence of ductal dilatation to be a common finding in BCCs; this is not the case for associations with real cysts. All the cases included in my series are large enough to be considered cysts without doubt.

In summary, I have presented the first and largest series of cases of collisions between BCC and ASFu tumors. I have reported three collisions that had not previously been described (infundibular cysts, steatocystomas, and a spiradenoma). Curiously, all trichilemmomas were associated with superficial BCC. I propose some factors that may influence the occurrence of collisions and explain the higher frequency of some collisions in comparison with others, opening up new lines of investigation.

## Figures and Tables

**Table 1:** Antibodies panel.

Antibody	Description	Laboratory	
SMA	Monoclonal mouse anti-human smooth muscle actin Clone 1A4	DAKO	Denmark
BerEP4	Monoclonal mouse anti-human epithelial antigen Clone Ber-EP4	DAKO	Denmark
CEA	Monoclonal mouse anti-human carcinoembryonic antigen Clone II-7	DAKO	Denmark
CK A1/A3	Monoclonal mouse anti-human cytokeratin Clone AE1/AE3	DAKO	Denmark
EMA	Monoclonal mouse anti-human epithelial membrane antigen Clone E29	DAKO	Denmark
Ki67	Monoclonal mouse anti-human Ki 67 antigen Clone MIB-1	DAKO	Denmark

SMA—alpha-smooth muscle actine.

**Table 2:** Epidemiology.

Case	Year ^+^	Sex/Age	Medical History	Immunosuppression	Comment
Case 1	2005	F/75	HBP, DM, Cardiopathy	NO	Exitus 2007
Case 2	2009	M/67	HBP, Prostatic sd, hypercholesterolemia, Hip prosthesis	NO *	Exitus 2012 due to lung carcinoma
Case 3	2009	F/85	Multinodular goiter, anxiety, multiple BCC	NO	
Case 4	2011	F/83	HBP, Hyperuricemia, Cardiopathy, Hypertriglyceridemia, BCC 2005	NO	
Case 5	2013	M/26	X. Pigmentosum, melanoma, multiple BCC, SCC, and KA	NO	
Case 6	2014	F/49	None	NO	
Case 7	2015	M/66	Nephrotic syndrome, immunosuppression treatment	YES	
Case 8	2016	F/66	BCC 2004	NO	
Case 9	2016	M/91	Prostatic cancer; Hodgkin lymphoma; BCC 2012	YES	
Case 10	2017	F/57	Primary biliary cirrhosis; Ulcerative colitis; Esophageal varices; Hypothyroidism;Breast cancer 2016; prednisone treatment	YES	Exitus 2017 due to breast cancer
Case 11	2017	M/66	BCC 2011	NO	
Case 12	2017	F/72	Arthrosis; Osteoporosis	NO	

^+^ year of diagnosis; * the patient died due to lung cancer 3 years after the diagnosis of collision. The diagnosis of lung cancer was made shortly after the occurrence of the collision; hence, the patient could have been in an immunosuppressed state; BCC —basal cell carcinoma; DM—diabetes mellitus; F—female; HBP—high blood pressure; KA—keratoacanthoma; M—male; SCC—squamous cell carcinoma; sd—syndrome; X—xeroderma.

**Table 3:** Histopathological and clinical data.

CASE Number	COLLISION		Location	Situation of the ASFu Neoplasm	Clinical Diagnosis
	BCC Type	ASFu Neoplasm			
Case 1	Nodular	Hidrocystoma	Upper lip	Below	BCC
Case 2	Nodular	Infundibular cyst	Left cheek	Below	BCC
Case 3	Adenoid	Steatocystoma	Right Retro auricular	Below	BCC
Case 4	Nodular/ Infiltrative	Spiradenoma	Forehead	Below	BCC
Case 5	Adenoid	Infundibular Cyst	Sternum	Below	BCC
Case 6	Nodular/Micronodular	Steatocystoma	Right cheek	Below	BCC
Case 7	Nodular	Infundibular cyst + Hidrocystoma	Nose	Intermingled	BCC
Case 8	Micronodular	Hidrocystoma	Nose	Below	BCC
Case 9	Micronodular	Clear-cell Hidradenoma	Back	Intermingled	BCC
Case 10	Superficial	Trichilemmoma	Right cheek	Intermingled	BCC
Case 11	Superficial	Trichilemmoma	Back	Intermingled	BCC
Case 12	Micronodular	Hidrocystoma	Forehead	Below	BCC vs. infundibular cyst

BCC—basal cell carcinoma; ASFu—apocrine–sebaceous–follicular unit.

**Table 4:** Immunochemistry.

Case	Neoplasm ASFu	BerEP4 BCC/ASFu	CEA BCC/ASFu	SMA BCC/ASFu	EMA BCC/ASFu	CKA1A3 BCC/ASFu	Ki67 BCC/ASFu	Giemsa BCC/ASFu
Case 1	Hidrocystoma	+/−	−/+ Luminal layer	+ stroma and focal/+ Outer layer				
Case 2	Infundibular cyst	+/−	−/−	+ focal/-				
Case 3	Steatocystoma	+/−	−/−	−/−				
Case 4	Spiradenoma	+/−	−/+ Ducts	+ stroma and focal/+ Peripheral layer and reticulate center	−/+ some cells			
Case 5	Infundibular cyst							
Case 6	Steatocystoma	+/−	−/+ Luminal layer	+ focal/+ erector pili muscle				
Case 7	Infundibular cyst + Hidrocystoma	+/−	−/− (Infundibular cyst) /+ Luminal layer hidrocystoma	+/+ outer layer/+ outer layer				
Case 8	Hidrocystoma	+/−	−/+ Luminal layer	−/+ outer layer				
Case 9	Clear-cell hidradenoma	+/+ solo cel claras	−/+ Ducts		−/+ in squamous cells	+/+	++/+	+/− stroma
Case 10	Trichilemmoma	+/+ focal						
Case 11	Trichilemmoma	+/+ weak focal			−/+ weak focal			
Case 12	Hidrocystoma	+/−	−/+ Ducts					

BCC—basal cell carcinoma; ASFu—apocrine–sebaceous–follicular unit.

## Data Availability

Data contained within the article. Additional data if available, can be shared upon reasonable request from any qualified investigator.
